# Template-dependent inhibition of coronavirus RNA-dependent RNA polymerase by remdesivir reveals a second mechanism of action

**DOI:** 10.1074/jbc.AC120.015720

**Published:** 2020-09-23

**Authors:** Egor P. Tchesnokov, Calvin J. Gordon, Emma Woolner, Dana Kocinkova, Jason K. Perry, Joy Y. Feng, Danielle P. Porter, Matthias Götte

**Affiliations:** 1 Department of Medical Microbiology and Immunology, University of Alberta, Edmonton, Alberta, Canada; 2 Gilead Sciences, Inc., Foster City, California, USA; 3 Li Ka Shing Institute of Virology, University of Alberta, Edmonton, Alberta, Canada

**Keywords:** COVID-19, coronavirus, CoV, SARS-CoV-2, RNA-dependent RNA polymerase, RdRp, replication, remdesivir, delayed chain termination, viral polymerase, drug development, drug resistance, RNA polymerase, RNA virus, SARS-2

## Abstract

Remdesivir (RDV) is a direct-acting antiviral agent that is used to treat patients with severe coronavirus disease 2019 (COVID-19). RDV targets the viral RNA-dependent RNA polymerase (RdRp) of severe acute respiratory syndrome coronavirus 2 (SARS–CoV-2). We have previously shown that incorporation of the active triphosphate form of RDV (RDV-TP) at position i causes delayed chain termination at position i + 3. Here we demonstrate that the S861G mutation in RdRp eliminates chain termination, which confirms the existence of a steric clash between Ser-861 and the incorporated RDV-TP. With WT RdRp, increasing concentrations of NTP pools cause a gradual decrease in termination and the resulting read-through increases full-length product formation. Hence, RDV residues could be embedded in copies of the first RNA strand that is later used as a template. We show that the efficiency of incorporation of the complementary UTP opposite template RDV is compromised, providing a second opportunity to inhibit replication. A structural model suggests that RDV, when serving as the template for the incoming UTP, is not properly positioned because of a significant clash with Ala-558. The adjacent Val-557 is in direct contact with the template base, and the V557L mutation is implicated in low-level resistance to RDV. We further show that the V557L mutation in RdRp lowers the nucleotide concentration required to bypass this template-dependent inhibition. The collective data provide strong evidence to show that template-dependent inhibition of SARS–CoV-2 RdRp by RDV is biologically relevant.

The U.S. Food and Drug Administration has recently issued an emergency use authorization (EUA) for the investigational drug remdesivir (RDV) to treat infection with severe acute respiratory syndrome coronavirus 2 (SARS–CoV-2) (1). The EUA was largely based on a randomized clinical trial that showed a significant reduction in the time of recovery of hospitalized individuals diagnosed with coronavirus disease 2019 (COVID-19) (2). RDV is a nucleotide analog prodrug that was designed to target the RNA-dependent RNA polymerase (RdRp) of RNA viruses (3, 4). The triphosphate form of RDV (RDV-TP) is an analog of the natural adenosine triphosphate (ATP) with 1′-C-nucleoside bond and a 1′-cyano-substitution. RDV has shown a broad-spectrum of antiviral activity in cell culture and animal models against negative-sense RNA viruses of the *Filoviridae* (*e.g.* Ebola virus) and *Paramyxoviridae* (*e.g.* Nipah virus and measles virus) as well as *in vitro* activity against viruses in the *Pneumoviridae* (*e.g.* respiratory syncytial virus) family (3–7). Antiviral activity against positive-sense RNA viruses, including flaviviruses and coronaviruses, was also demonstrated in various systems (8–12). Several previous studies have demonstrated prophylactic and therapeutic efficacy in animal models of SARS-CoV, Middle East respiratory syndrome (MERS)-CoV and SARS–CoV-2 (10, 12).

Low 50% effective concentrations (EC_50_) and a high barrier to the development of resistance were observed in cell cultures (8). EC_50_ values in the lower nanomolar range have been measured for each of these three viruses, depending on the cell type–specific metabolism of RDV-TP (12). *In vitro* selection experiments with the mouse hepatitis virus (MHV) revealed two mutations in the RdRp enzyme that confer low-level resistance to RDV (8). F476L and V553L were shown to cause 2.4-fold resistance and 5.0-fold resistance, respectively. The F476L+V553L double mutant showed 5.5-fold resistance to RDV. The equivalent mutations F480L and V557L introduced together in SARS-CoV were also shown to confer low-level (6.0-fold) resistance to the drug (8). Resistance data for SARS–CoV-2 are pending.

Despite progress, there are gaps in our understanding of the mechanism of action of RDV. A key element of a refined mechanism of action is based on high rates of incorporation of RDV-TP, which translates in efficient competition with its natural counterpart ATP (13, 14). However, the existence of a 3′-OH group allows further nucleotide incorporation events to occur and inhibition of RNA synthesis is not immediately evident. Biochemical experiments with various recombinant RdRp complexes from both minus-sense and plus-sense RNA viruses show delayed chain termination (5, 6, 13, 14). RdRp complexes of SARS-CoV, MERS-CoV, and SARS–CoV-2 stop RNA synthesis after position i + 3, *i.e.* three nucleotides following incorporation of RDV-TP at position i (13, 14). This pattern is identical with the three RdRp complexes, which points to a common mechanism of inhibition. Modeling studies suggested that a steric clash between the side chain of the conserved Ser-861 and the 1′-CN group of the incorporated RDV prevent translocation, which means that the nucleotide binding site at position i + 4 is still occupied with the 3′-end of the primer (14). Another important observation is that RNA synthesis arrest can be overcome with higher concentrations of natural nucleotide pools (14). Increasing the NTP concentrations gradually reduces termination efficiency and favors read-through to yield the full-length RNA product. Efficient read-through is seen at concentrations as low as 10 μm. Intracellular NTP concentrations are in the high μm and low mm range (15, 16), which suggests that read-through reactions likely occur under biologically relevant conditions. It is therefore conceivable that the primer strand, and by extension the entire negative-sense copy of the genome, contains several RDV residues. Although the CoV exonuclease (ExoN) exhibits 3′–5′ proofreading activity (8, 17–19), access to internal RDV residues is expected to be compromised. This scenario raises the question whether RDV residues embedded in the template strand may also cause inhibition during synthesis of the second RNA strand, *i.e.* during transcription or viral genome synthesis.

To address this question, we have devised a method that allows synthesis of small RNA model templates with RDV-TP incorporated at a single strategic position. RNA synthesis with recombinant SARS–CoV-2 RdRp shows that incorporation of UTP opposite RDV and the following nucleotide incorporation are compromised. Similar to earlier observations with the primer strand, this inhibition can be overcome with increasing NTP concentrations, although the threshold is much higher here. This effect is partially reversed with a known resistance-conferring mutation that can affect the positioning of the template strand. Based on these data, we propose a comprehensive mechanism of action of RDV that involves both the primer strand and the template.

## Results

### Effects of mutations at position Ser-861 on delayed chain termination

Several recent structural and biochemical studies have demonstrated that the active SARS–CoV-2 RdRp complex is composed of the three nonstructural proteins nsp7, nsp8, and nsp12 (20–24). Reconstitution of separately expressed proteins commonly yields complexes with stoichiometry of 1:1:2 for the nsp12 (RdRp) and the other components nsp7 and nsp8, respectively. We utilized constructs that co-express the viral protease nsp5 together with nsp7, nsp8, and nsp12 in insect cells (13, 14). Active complexes were captured via the histidine-tagged nsp8, and binary complexes composed of nsp8 and nsp12 were identified with Coomassie Blue staining. Here we show that overloading of the gel with the soluble protein sample also visualizes nsp7 (Fig. S1). Using the same construct design, we generated complexes with mutations at position Ser-861 in nsp12 that are expected to affect efficiency of delayed chain termination with RDV-TP. The steric clash hypothesis predicts that smaller side chains will reduce delayed chain termination and larger side chains will increase inhibition, provided that substitutions do not impede regular NTP incorporations.

**Figure 1.**
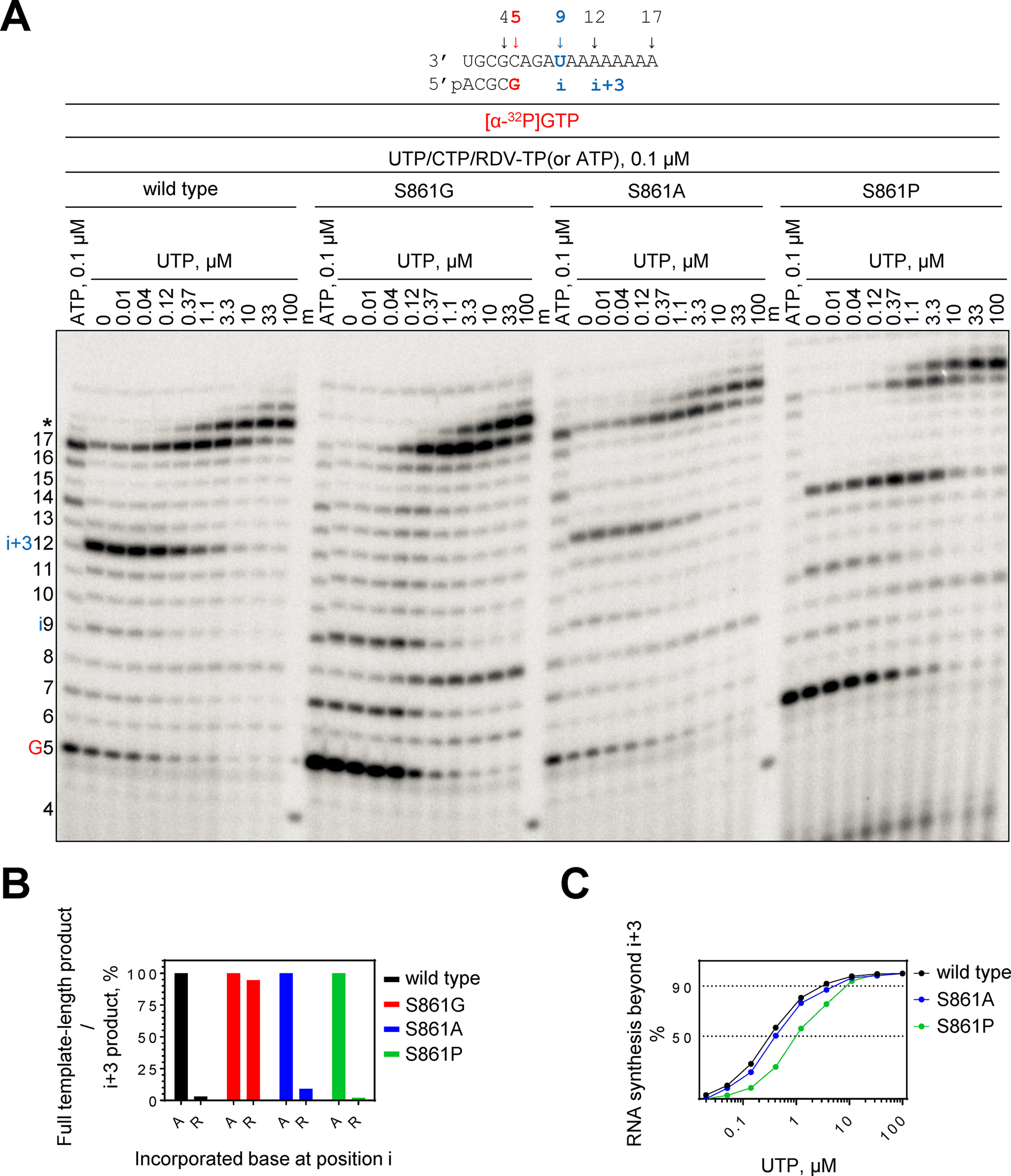
**RNA synthesis catalyzed by SARS–CoV-2 RdRp WT, S861G, S861A, and S861P mutant complexes on an RNA template containing single U for RDV-TP incorporation.** *A*, RNA primer/template supporting incorporation of RDV-TP at position 9 (i = 9) is shown on top. i + 3 illustrates delayed chain termination at position 12. G5 indicates incorporation of [α-^32^P]GTP at position 5. RNA synthesis was catalyzed by SARS–CoV-2 RdRp WT and mutant complexes in the presence of RNA primer/template, MgCl_2_, and indicated concentrations of NTP and RDV-TP for 30 min. Reactions loaded in lanes *ATP, 0.1* μ*m* contained only UTP/CTP/ATP at 0.1 μm; reactions loaded in the remaining lanes contained UTP/CTP/RDV-TP, each at 0.1 μm, supplemented with indicated concentrations of UTP. *4* indicates the migration pattern of 5′-^32^P-labeled 4-nt primer used here as a size marker. *Asterisk* indicates slippage because of the poly(A) context of the template. *B*, graphic representation of the effect of delayed chain termination at i + 3 on full template-length RNA synthesis. The fold-ratio of full-length product 17 to delayed chain termination at i + 3 when RDV-TP is present in the reaction (in the absence of supplemental UTP). This value was normalized to the corresponding ratio when ATP was present in the reaction. Normalized values were plotted *versus* incorporated nucleotide at position i. *C*, graphic representation of products of RNA synthesis beyond position i + 3 plotted as a function of UTP concentration.

We compared RNA synthesis of the WT SARS–CoV-2 RdRp complex with S861G, S861A, and S861P mutants (Fig. 1). Reactions were monitored on a 17-mer RNA template that contains a single site of incorporation for RDV-TP at position 9 or i (Fig. 1*A*). The WT enzyme shows delayed chain termination at position 12 (i + 3) (Fig. 1*A*), which is illustrated by more than 95% reduction in the full template length-to–(i + 3) product ratio in the presence of RDV-TP as compared with the full template length-to–p12 product ratio in the presence of ATP (Fig. 1*B*). Termination efficiency is gradually reduced by increasing the concentration of the next incoming nucleotide (UTP), as previously reported (14). 50% read-through is seen at UTP concentrations of ∼0.3 μm, 90% read-through is seen with ∼3 μm, and inhibition is abolished with ∼30 μm (Fig. 1*C*). Delayed chain termination is not observed with the S861G mutant. These findings are consistent with the removal of a steric clash when serine is replaced with glycine. Full-length product formation is already seen to near completion at UTP concentrations of ∼1 μm (Fig. 1*A*). The S861A mutation shows only subtle reductions in delayed chain termination (Fig. 1, *A* and *B*), which is in agreement with a previous report by Wang and colleagues (23). We further show that the bulkier side chain of S861P shows subtle increases in UTP concentrations required to overcome delayed chain termination (Fig. 1, *A* and *C*).

### RDV inhibits RNA synthesis when embedded in the template

**Figure 2.**
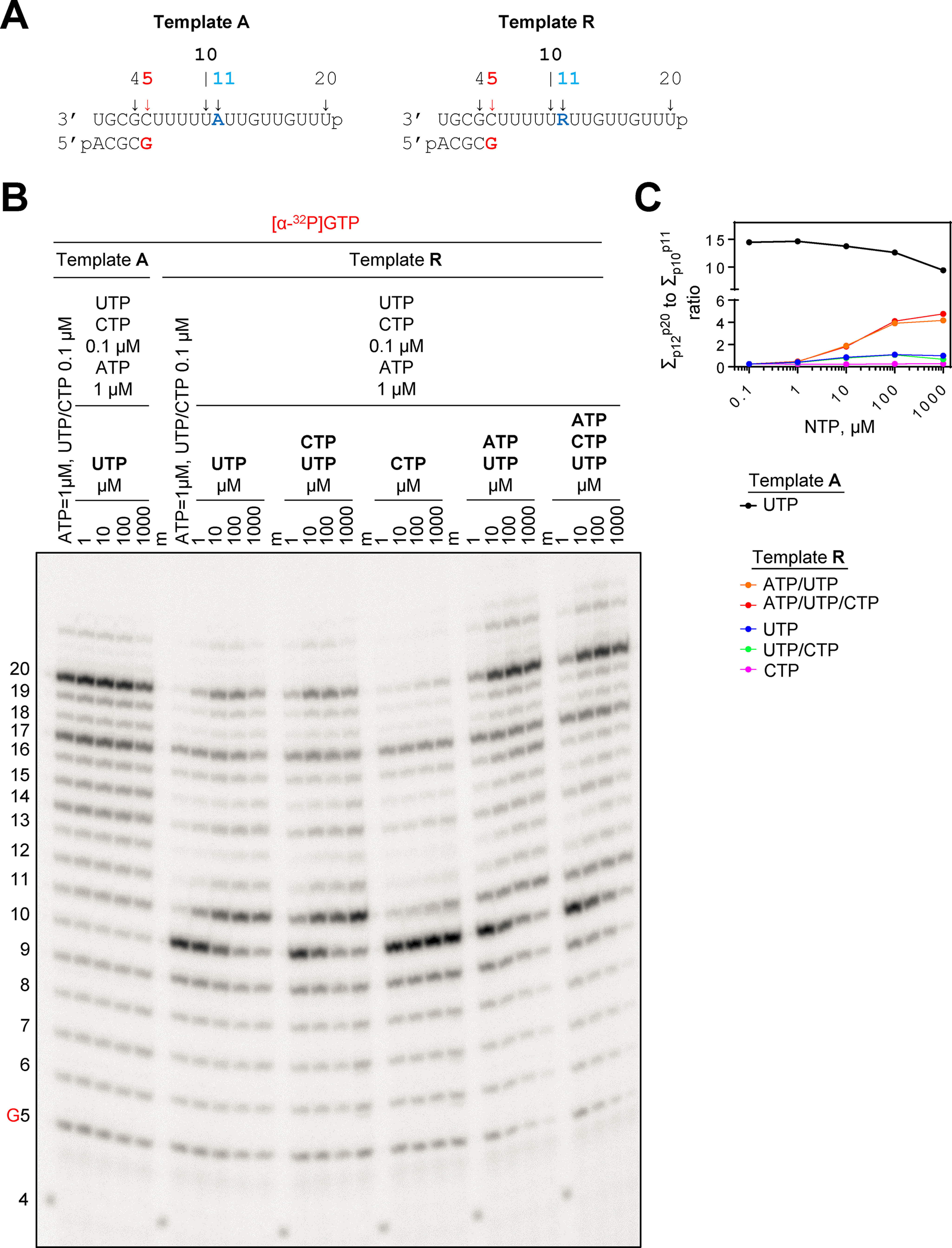
**RNA synthesis catalyzed by SARS–CoV-2 RdRp using a template with a single RDV residue at position 11.** *A*, RNA primer/template with template-embedded RDV (*Template R*) at position 11; the corresponding primer/template (*Template A*) with adenosine at this position is shown on the *left*. G5 indicates the incorporation of [α-^32^P]GTP opposite template position 5. *B*, migration pattern of the products of RNA synthesis catalyzed by SARS–CoV-2 RdRp WT complex in the presence of RNA primer/templates shown in *panel A*, MgCl_2_, and indicated concentrations of NTP cocktails after incubation for 30 min. *4* indicates the migration pattern of 5′-^32^P-labeled 4-nt primer used here as a size marker. *C*, graphic representation of the fold-ratio of the sum of products 12 through 20 to the sum of products 10 and 11.

The high efficiency of read-through with increasing NTP concentrations suggests that the newly synthesized copy of the RNA genome contains several RDV residues. This raises the question whether RDV may also inhibit RNA synthesis when present in the template (Fig. 2). The challenge to synthesize sufficient amounts of RNAs with embedded RDV residues using RdRp is the need for strand separation. Hence, we developed a protocol to generate single-stranded RNAs using T7 RNA polymerase along a DNA template that can be selectively degraded (see “Experimental procedures”). We synthesized two 20-mer model RNAs that contain either adenosine (*Template A*) or RDV (*Template R*) at the same strategic position 11 (Fig. S2).

A 4-mer primer was used to initiate RNA synthesis with SARS–CoV-2 RdRp in the presence of [α-^32^P]GTP (Fig. 2*A*). With Template A, a mixture of minimal concentrations of 0.1 μm CTP and UTP, and 1 μm ATP yields predominantly the full-length 20-mer RNA product (Fig. 2*B*). Increasing the concentration of UTP shows no significant increases in RNA synthesis. With Template R, we observe an intermediate product at position 10 at the same base concentrations of CTP, UTP, and ATP (Fig. 2*B*). These findings demonstrate that 0.1 μm UTP is not sufficient for incorporation opposite RDV, which is a marked difference to results obtained with Template A. Increasing concentrations of UTP can overcome the inhibitory effects, and we observe an accumulation of product at position 11, which points to a second site of inhibition immediately after the template-embedded RDV. A similar effect is seen with increasing the concentration of UTP and CTP that is required later at position 14. Increases only in CTP did not yield significant amounts of product at position 11, suggesting that incorporation of CTP opposite RDV is negligible. Higher concentrations of UTP and ATP or UTP, ATP, and CTP gradually reduced the inhibitory effects and increased the yield of full-length product. Overall inhibition is driven by the accumulation of products at positions 10 and 11. Together these findings show that a single RDV residue in the template inhibits efficiency of incorporation of the complementary UTP and the adjacent NTP (ATP in this particular sequence context). NTP concentrations required to overcome these obstacles are higher than observed with delayed chain termination (Fig. 2*C*).

### The V557L mutation in nsp12 counteracts the inhibitory effects of RDV in the template

**Figure 3.**
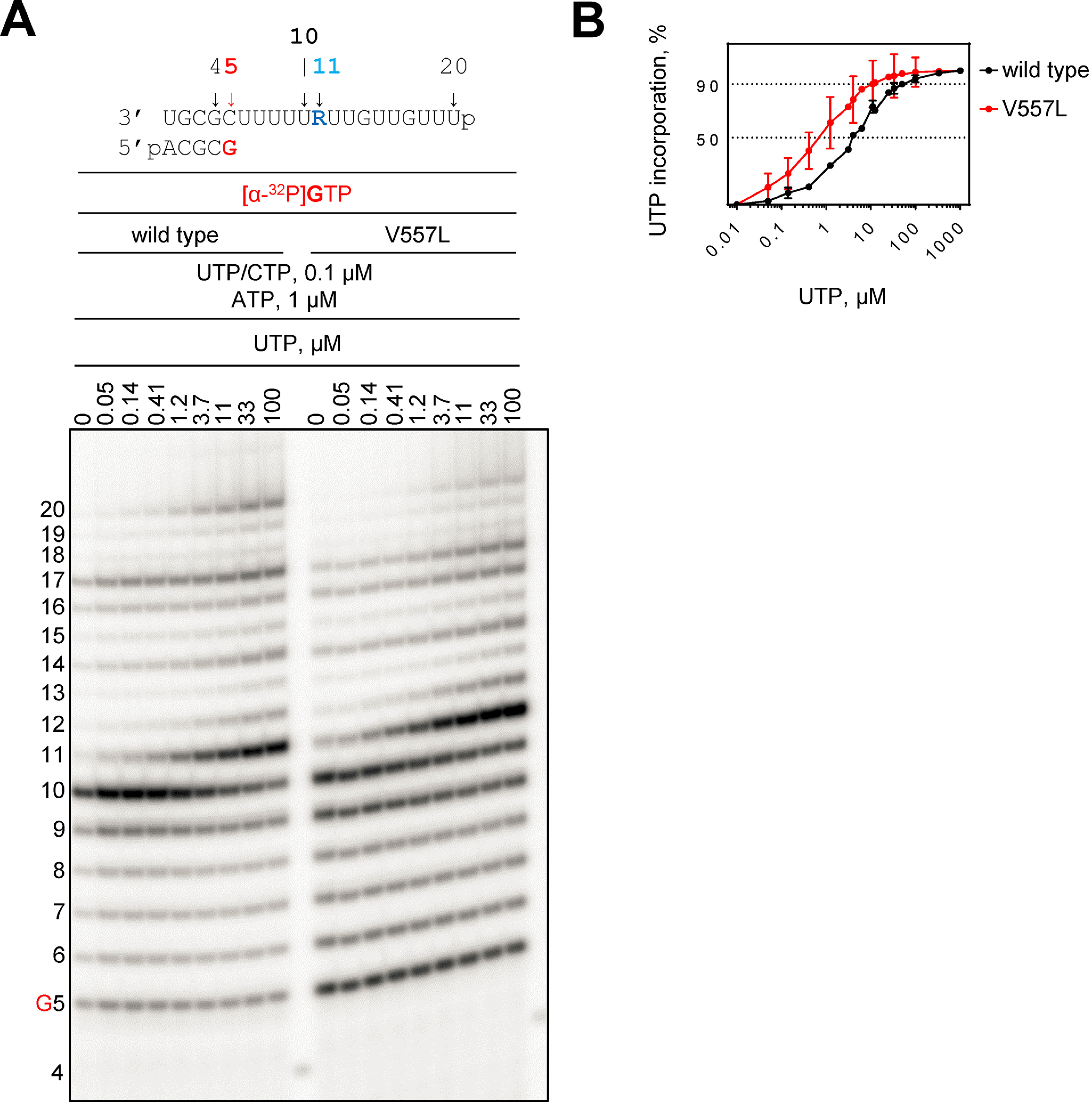
**RNA synthesis catalyzed by SARS–CoV-2 RdRp WT and the V557L mutant complex on Template R.** *A*, RNA primer/template with template-embedded RDV at position 11 is shown on top. G5 indicates the incorporation of [α-^32^P]GTP opposite template position 5. Below the primer/template sequence is the migration pattern of the products of RNA synthesis catalyzed by SARS–CoV-2 RdRp complexes in the presence of RNA primer/template, MgCl_2_, indicated concentrations of NTP mixture supplemented with indicated concentrations of UTP after 30 min. *4* indicates the migration pattern of 5′-^32^P-labeled 4-nt primer used here as a size marker. *B*, graphic representation of the percent UTP incorporation opposite RDV plotted as a function of UTP concentration.

We next asked whether such template-dependent inhibition provides a biologically relevant mechanism of action of RDV. To address this question we attempted to study possible neutralizing effects of known resistance conferring mutations. In nsp12, two amino acid substitutions have been associated with low-level resistance to RDV (8). The effect of F480L is subtle and structural data do not suggest a direct effect on RNA synthesis. In contrast, the hydrophobic side chain of Val-557 is located close to the template opposite the incoming nucleotide (12). Hence, a mutation at this position could conceivably affect nucleotide incorporation. We therefore expressed and purified the V557L mutant and studied its potential effect on the concentration of UTP required to overcome the obstacle imposed by the complementary RDV (Fig. 3). We compared WT RdRp with the V557L mutant. Increasing the concentrations of UTP gradually from 0.1 μm to 100 μm showed the following effects (Fig. 3*A*). The primary observation is that the UTP concentration required to overcome inhibition at position 10 and to generate the product at position 11 is lower with the Val-557 mutant (0.7 *versus* 4 μm for 50% incorporation and 10 *versus* 50 μm for 90% incorporation of UTP by V557L and WT, respectively) (Fig. 3*B*). The associated steady-state kinetic parameters show that UTP incorporation is ∼5-fold more efficient with the mutant enzyme (Table S1). The effect of V557L is specific to incorporation of UTP opposite RDV. Incorporation of the next nucleotide (ATP) is not significantly affected by the V557L mutant enzyme (Fig. S3 and Table S1).

However, the increase in efficiency of UTP usage with the V557L mutant does not translate in increases in the amount of the full-length product (Fig. 3*A*). These findings suggest that regular nucleotide incorporations might be compromised, which is not unusual for enzymes that contain resistance-conferring mutations. Steady-state kinetic parameters show that the efficiency of ATP incorporation is ∼3-fold reduced with the mutant enzyme (Table S2). However, it is important to note that the selectivity (*V*_max_/*K_m_* (ATP)/*V*_max_/*K_m_* (RDV-TP)) is almost identical for V557L and the WT enzyme. Thus, V557L does not show a resistance phenotype at the level of incorporation of RDV-TP. Moreover, V557L is also not significantly different from the WT in assays monitoring delayed chain termination with either single or multiple sites of incorporation for RDV-TP (Fig. S4). The collective data provide evidence to show that the effect of V557L is specific to RDV embedded in the template, which in turn suggests that this type of inhibition is biologically relevant. Moreover, the S861G mutant specifically reduces delayed chain termination and does not affect template-dependent inhibition of RNA synthesis (Fig. S5). However, this mutation is here newly described, and it remains to be seen whether the corresponding virus is viable and may affect drug susceptibility.

**Figure 4.**
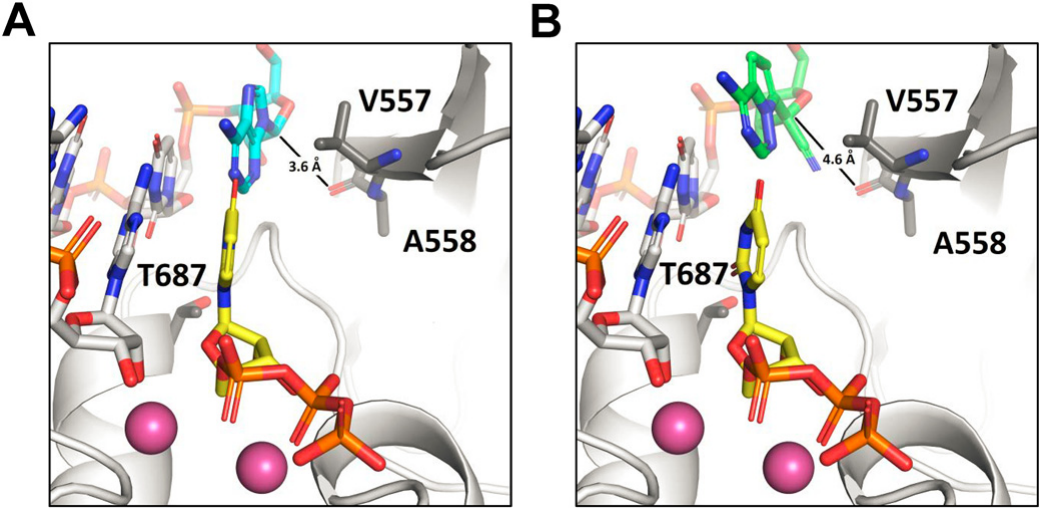
*A* and *B*, structural model of UTP incorporation with (*A*) adenosine (*cyan*) serving as the template base and (*B*) RDV (*green*) serving as the template base. With respect to the optimal position seen with adenosine, RDV is significantly perturbed, shifting by ∼1 Å, because of a clash between its 1′-CN and the protein backbone at residue Ala-558.

**Figure 5.**
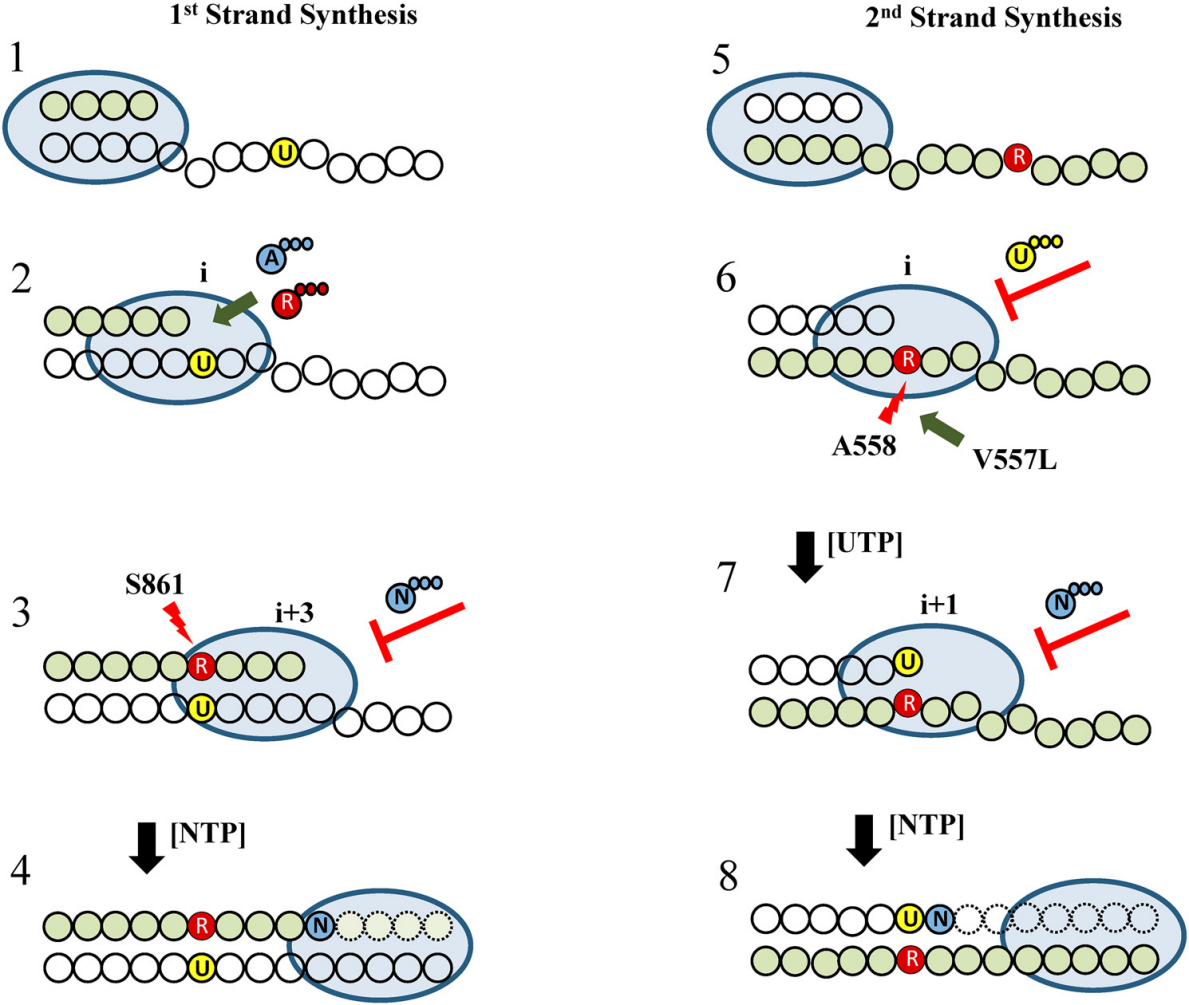
**Model of the mechanism of action of RDV against SARS–CoV-2 RdRp.** *Left*, a model for inhibition during synthesis of the first RNA strand is adapted from our previous study (14). The priming strand is shown with *green* circles, *colorless* circles represent residues of the template, and the *light blue* oval represents the RdRp complex (*stage 1*). RDV-TP (*R*, *red*) competes with ATP (*A*, *blue*) for incorporation opposite template *U* labeled *yellow* (*stage 2*). RNA synthesis is terminated after the addition of three more nucleotides. This effect is referred to as delayed chain termination as a result of a steric clash between Ser-861 (*red arrow*) and the 1′-CN group of the incorporated RDV (*stage 3*). Higher concentrations of NTP can overcome this effect and read-through facilitates formation of full-length RNA products (*stage 4*). This mechanism retains RDV residues in the primer strand that is later used as a template (*stage 5*). Incorporation of UTP opposite RDV is compromised because of a steric clash between Ala-558 (*red arrow*) and RDV's 1′-CN group (*stage 6*). Higher concentrations of the next nucleotide can overcome inhibition. This concentration is further reduced with the resistance-associated Val-557 mutant enzyme (*green arrow*). NTP incorporation at the adjacent position is likewise inhibited (*stage 7*). This obstacle is also partially overcome with higher NTP concentrations (*stage 8*).

### Modeling shows template RDV is significantly perturbed relative to A

Building on our previous models of the pre-incorporation state of the replicating SARS–CoV-2 nsp7/nsp8/nsp12 complex (12, 14), we found that RDV, when serving as template for the incoming UTP, is not properly positioned (Fig. 4). In the absence of any relaxation relative to the reference A-containing template, the 1′-CN has a significant clash with the backbone carbonyl of residue Ala-558. Allowing for minimization of RDV and the template overhang, the clash can be relieved, but base pairing between RDV and UTP is compromised. As a result, RDV functions as a poor template for the incoming NTP. Furthermore, translocation to the next template position may involve additional clashes, particularly between the 1′-CN and residue Thr-687. This model therefore provides a plausible explanation for data shown in Fig. 2. However, the structural reasons for V557L resistance remain elusive and a fuller dynamic examination or structural data may be required.

## Discussion

The investigational nucleotide analog prodrug RDV is a direct-acting antiviral that has been approved in several countries for the treatment of COVID-19 patients (25). Previously, we initiated studies on the mechanism of action of RDV and demonstrated that RDV-TP is efficiently incorporated into the growing RNA chain by recombinant SARS–CoV-2 RdRp (14). Inhibition of RNA synthesis is delayed and observed at position i + 3, *i.e.* three residues downstream of the site of incorporation. However, higher concentrations of NTP pools can reduce the efficiency of delayed chain termination, concomitantly enhancing read-through and full-length product formation (13, 14). Efficient read-through may therefore yield a complete copy of the positive-sense RNA genome with embedded RDV residues. This negative-sense RNA is then utilized as a template for viral genome synthesis and the generation of RNA transcripts. It is currently unknown whether RDV exhibits inhibitory effects when present in the template. Using a biochemical approach, we have addressed this question directly and propose a model of the mechanism of action of RDV against SARS–CoV-2 RdRp that involves both RNA strands (Fig. 5).

The mechanism of inhibition during synthesis of the first RNA strand is adapted from our previous study (14). In this model, RNA synthesis by SARS–CoV-2 RdRp is reduced to the polymerase, the primer strand, and the template. The complex interactions between nsp7, nsp8, nsp12, RNA, and other viral factors that are implicated in replication and transcription are not considered (stage 1). As RNA synthesis proceeds, RDV-TP competes with its natural counterpart ATP for incorporation opposite template U at a random position “i” (stage 2). Steady-state kinetics suggests that the efficiency of incorporation is three times higher with RDV-TP (13, 14), which is unusually efficient for a nucleotide analog inhibitor. The presence of a 3′-hydroxyl group allows a nucleophilic attack on the next incoming nucleotide and no significant inhibition is seen at position i + 1. Three additional nucleotides are incorporated before RNA synthesis is arrested at position i + 3 (stage 3). Inhibitors of this type are commonly referred to as “delayed chain terminators,” in contrast to obligate or classic chain terminators that lack the 3′-hydroxyl group and prevent further nucleotide additions at i + 1 (26). Modeling suggested that a steric clash between the side chain of Ser-861 and the 1′-CN group of RDV blocks translocation of the RdRp complex into position i + 4. This hypothesis was supported by experiments with the S861A mutant that showed subtle reductions in efficiency of delayed chain termination (23). Here we demonstrate that the smaller S861G eliminates termination.

At this stage it is important to note that the steric clash does not represent an unsurmountable obstacle. Delayed chain termination is gradually reduced when increasing the concentration of the next incoming nucleotide. This effect is also shown here even with the bulkier S861P substitution. The reduction in termination efficiency coincides with an increase in read-through monitored as full-length RNA product synthesis. A possible explanation is that the increased NTP concentration shifts the translocational equilibrium from i + 3 (pre-translocation) to i + 4 (post-translocation), which liberates the nucleotide binding site. For HIV type 1 (HIV-1) reverse transcriptase (RT), we developed site-specific footprinting techniques that demonstrated such nucleotide-dependent translocation (28). Seifert and colleagues (29) recently applied a single molecule approach, based on magnetic tweezers, to study the function of SARS–CoV-2 RdRp and its inhibition with nucleotide analogues. The authors found that the incorporated RDV causes enzyme pausing at high NTP concentrations, whereas termination was not observed. The full-length products seen in this study are equivalent to read-through events and full-length product formation seen in our experiments. It is therefore tempting to predict that the S861G mutant will eliminate or reduce pausing. Each of the aforementioned studies provide evidence to suggest that synthesis of the first RNA strand may continue to completion especially in the presence of high concentrations of NTPs. We determined that NTP concentrations as low as 10 μm cause ∼90% read-through.

The 3′–5′ proofreading ExoN activity of the replication complex plays another important role in the generation of RNA strands with embedded RDV residues. ExoN(−) MHV shows an ∼5-fold increase in RDV susceptibility; however, potent inhibition was still seen with an intact ExoN (8). This partial protection from 3′–5′ proofreading may therefore preserve RDV residues in complete copies of the first RNA strand (stage 5). This assumption provides the basis for a template-dependent inhibition mechanism. Here we show that incorporation of UTP at position i opposite RDV is indeed compromised. UTP concentrations of ∼50 μm are required to bypass this obstacle (stage 6). This concentration is significantly lowered with the V557L mutant, which provides a mechanism for drug resistance (8). Val-557 is located in close proximity to the incorporated RDV. It is therefore conceivable that the V557L facilitates incorporation of UTP through repositioning of the template strand. The effect of V557L is specifically seen when RDV is present in the template. WT and V557L mutant do not show significant differences regarding selective incorporation of RDV-TP or delayed chain termination.

The link between the newly discovered template-dependent inhibition and a resistance-conferring mutation suggests that this mechanism is biologically relevant. However, it is important to note that neither V557L nor F480L or any other RDV resistance–associated mutations in SARS–CoV-2 have been reported at this point. The selection of the structural equivalent V553L MHV required a high number of passages and RNA levels in the presence of drug are still lower than RNA levels in the absence of drug (8). These results indicate a high barrier to the development of resistance, which is likely because of a fitness deficit associated with this mutation. In agreement with these observations, we demonstrate the V557L in SARS–CoV-2 RdRp diminishes efficiency of regular nucleotide incorporations and neutralizes its advantageous effects on UTP incorporation opposite RDV. Hence, the barrier to the selection of V557L might be even higher for SARS–CoV-2. Moreover, incorporation of the adjacent NTP, immediately downstream of the newly added UTP is likewise inhibited and the effect of V557L is less pronounced at this position (stage 7). NTP concentrations required to overcome this obstacle exceed 100 μm and rescue of RNA synthesis remains incomplete even at higher NTP concentrations (stage 8). Finally, we note that template-dependent inhibition mechanisms have also been demonstrated with other nucleotide analog inhibitors (26, 30–32).

In conclusion, RDV can inhibit RNA synthesis by SARS–CoV-2 RdRp when incorporated in the primer strand and when present in the template strand. The two distinct modes of inhibition can be linked to interactions between the 1′-CN group of the nucleotide analog and conserved residues in nsp12. The side chain of Ser-861 causes a steric clash with the incorporated RDV and inhibition is seen at position i + 3. The backbone of Ala-558 causes steric problems when UTP is incorporated opposite RDV in the template and inhibition is seen at positions i and i + 1. The neighboring resistance-associated mutation V557L counteracts this effect. The challenge is to determine which mechanism is dominant. Delayed chain termination is solely seen at low concentrations of NTPs; however, the highly efficient use of RDV-TP can lead to multiple incorporation events, *e.g.* along polyU-tracts, that can amplify inhibition (Fig. S4). This effect has been demonstrated with various RdRp complexes (5, 6, 14, 33). Template-dependent inhibition can be observed at higher NTP concentrations, but this mechanism relies on a certain degree of protection from proofreading that retains RDV in copies of the viral genome. Biochemical approaches often do not capture the complexity of antiviral effects in a cellular environment. It is therefore important to continue to monitor the emergence of resistance in attempts to link specific amino acid substitutions to specific inhibition pathways. This work helps to guide drug development efforts and the design of future structural or kinetic studies that address these problems.

## Experimental procedures

### Nucleic acids and chemicals

RNA primers and templates, except RNA templates with embedded RDV, used for in this study were 5′-phosphorylated and purchased from Dharmacon (Lafayette, CO, USA). RDV-TP was provided by Gilead Sciences (Foster City, CA, USA). NTPs were purchased from GE Healthcare. [α-^32^P]GTP was purchased from PerkinElmer.

### Protein expression and purification

SARS–CoV-2 RdRp WT and mutant (V557L, S861A, S861G, and S861P) were expressed and purified as we reported previously (13, 14). Additional details on the constructs, protein expression and purification are also provided in supporting information.

### Evaluation of RNA synthesis and inhibition studies

RNA synthesis assays involving RDV-TP incorporation by SARS–CoV-2 RdRp complex, data acquisition and quantification were done as previously reported by us (27, 33, 34). Final enzyme concentrations in the reaction mixtures were 0.15 μm for WT, S861A, S861G, and S861P mutants, and 0.45 μm for the V557L mutant.

### Production of a model RNA template with an embedded remdesivir residue

Our initial tests revealed that T7 RNA polymerase (Thermo Fisher Scientific) readily accepted short RNA/DNA primer/templates for RNA synthesis and was capable of incorporating RDV-TP at high concentrations of 100 μm followed by a full template-length RNA synthesis (data not shown). The following DNA template (Dharmacon, Lafayette, CO, USA) was used as the starting material: 3′-AAACAACAA**T**AAAAAGCGCA-5′. *Underlined* portion indicates the region which is complementary to the 5′-monopyosphorylated RNA primer: 5′-pUUUGUUGUU. The template residue “**T**” indicates the site of the RDV-TP incorporation into the RNA primer. Therefore, the fully extended primer will contain an embedded remdesivir (**R**) and may serve as a 20-nt RNA template for the RNA synthesis by SARS–CoV-2 RdRp complex: 5′-pUUUGUUGUU**R**UUUUUCGCGU-3′, where underlined portion is complementary to the RNA primer 5′-pACGC used in SARS–CoV-2 RNA synthesis reactions. Note that this RNA primer can only anneal to a fully synthesized 20-nt RNA template. The T7 RNA polymerase reaction mixtures contained 30 units of the polymerase, 200 μm RNA primer, 100 μm DNA template, 100 μm UTP/CTP/GTP mixture, and 100 μm ATP or RDV-TP in a 25 mm Tris-HCl buffer (pH 8). Reactions were started with 5 mm MgCl_2_, incubated at 37°C for 90 min, boiled for 10 min, and incubated with 2 units of Turbo DNase (Thermo Fisher Scientific) at 37°C for 30 min. The reaction mixtures were then extracted with phenol/chlorophorm (premixed with isoamyl alcohol 25:24:1; BioShop, Burlington, ON, Canada) and buffer-exchanged consecutively three times using the size-exclusion chromatography Bio-Rad p6 spin columns (Bio-Rad Laboratories).

### Evaluation of RNA synthesis across the RNA template with embedded RDV

RNA synthesis assays by SARS–CoV-2 RdRp complex on an RNA template with embedded remdesivir (5′-pUUUGUUGUU**R**UUUUUCGCGU-3′, where *underlined* portion is complementary to the RNA primer 5′-pACGC), or with adenosine at the equivalent position (5′-pUUUGUUGUU**A**UUUUUCGCGU-3′), data acquisition and quantification were done as previously reported by us (27, 33, 34) with the following adjustments: reaction mixtures contained ∼5 μm RNA template, and enzyme concentrations were increased to ∼1 μm for the WT and ∼1.5 μm for the V557L mutant RdRp complexes to favor the full template-length RNA synthesis. Five independent preparations of RDV-embedded RNA templates and at least three independent preparations of SARS–CoV-2 WT and V557L mutant enzymes were used in this study.

### Structural modeling

Modeling was done following the general approaches outlined previously (12, 14). A major update to the model was done by using the more recent and more complete cryo-EM structure of Hillen et al. (22) (PDB ID: 6YYT). The pre-incorporation state incorporated elements from the previous models into this new structure, with optimization achieved via minimization and conformational searches done with the Schrödinger suite of programs (Schrödinger Release 2020-2: Prime, Schrödinger, LLC, New York, NY).

## Data availability

All data are included within this article.

## Supplementary Material

Supporting Information

## References

[1] U.S. Food and Drug Administration. (2020) *Fact Sheet for Health Care Providers Emergency Use Authorization (EUA) of Veklury*^®^ (*remdesivir*), pp. 1–20. Food and Drug Administration, Silver Spring, MD

[2] Wang Y., Zhang D., Du G., Du R., Zhao J., Jin Y., Fu S., Gao L., Cheng Z., Lu Q., Hu Y., Luo G., Wang K., Lu Y., Li H., et al (2020) Remdesivir in adults with severe COVID-19: A randomised, double-blind, placebo-controlled, multicentre trial. Lancet 395, 1569–1578 10.1016/S0140-6736(20)31022-9 32423584 PMC7190303

[3] Lo M. K., Jordan R., Arvey A., Sudhamsu J., Shrivastava-Ranjan P., Hotard A. L., Flint M., McMullan L. K., Siegel D., Clarke M. O., Mackman R. L., Hui H. C., Perron M., Ray A. S., Cihlar T., et al (2017) GS-5734 and its parent nucleoside analog inhibit Filo-, Pneumo-, and Paramyxoviruses. Sci. Rep. 7, 43395 10.1038/srep43395 28262699 PMC5338263

[4] Siegel D., Hui H. C., Doerffler E., Clarke M. O., Chun K., Zhang L., Neville S., Carra E., Lew W., Ross B., Wang Q., Wolfe L., Jordan R., Soloveva V., Knox J., et al (2017) Discovery and synthesis of a phosphoramidate prodrug of a pyrrolo[2,1-f][triazin-4-amino] adenine c-nucleoside (GS-5734) for the treatment of Ebola and emerging viruses. J. Med. Chem. 60, 1648–1661 10.1021/acs.jmedchem.6b01594 28124907

[5] Warren T. K., Jordan R., Lo M. K., Ray A. S., Mackman R. L., Soloveva V., Siegel D., Perron M., Bannister R., Hui H. C., Larson N., Strickley R., Wells J., Stuthman K. S., Van Tongeren S. A., et al (2016) Therapeutic efficacy of the small molecule GS-5734 against Ebola virus in rhesus monkeys. Nature 531, 381–385 10.1038/nature17180 26934220 PMC5551389

[6] Jordan P. C., Liu C., Raynaud P., Lo M. K., Spiropoulou C. F., Symons J. A., Beigelman L., and Deval J. (2018) Initiation, extension, and termination of RNA synthesis by a paramyxovirus polymerase. PLoS Pathog. 14, e1006889 10.1371/journal.ppat.1006889 29425244 PMC5823471

[7] Lo M. K., Feldmann F., Gary J. M., Jordan R., Bannister R., Cronin J., Patel N. R., Klena J. D., Nichol S. T., Cihlar T., Zaki S. R., Feldmann H., Spiropoulou C. F., and de Wit E. (2019) Remdesivir (GS-5734) protects African green monkeys from Nipah virus challenge. Sci. Transl. Med. 11, eaau9242 10.1126/scitranslmed.aau9242 31142680 PMC6732787

[8] Agostini M. L., Andres E. L., Sims A. C., Graham R. L., Sheahan T. P., Lu X., Smith E. C., Case J. B., Feng J. Y., Jordan R., Ray A. S., Cihlar T., Siegel D., Mackman R. L., Clarke M. O., et al (2018) Coronavirus susceptibility to the antiviral remdesivir (GS-5734) is mediated by the viral polymerase and the proofreading exoribonuclease. mBio 9, e00221–18 10.1128/mBio.00221-18 29511076 PMC5844999

[9] Brown A. J., Won J. J., Graham R. L., Dinnon K. H. 3rd, Sims A. C., Feng J. Y., Cihlar T., Denison M. R., Baric R. S., and Sheahan T. P. (2019) Broad spectrum antiviral remdesivir inhibits human endemic and zoonotic deltacoronaviruses with a highly divergent RNA dependent RNA polymerase. Antiviral Res. 169, 104541 10.1016/j.antiviral.2019.104541 31233808 PMC6699884

[10] de Wit E., Feldmann F., Cronin J., Jordan R., Okumura A., Thomas T., Scott D., Cihlar T., and Feldmann H. (2020) Prophylactic and therapeutic remdesivir (GS-5734) treatment in the rhesus macaque model of MERS-CoV infection. Proc. Natl. Acad. Sci. U. S. A. 117, 6771–6776 10.1073/pnas.1922083117 32054787 PMC7104368

[11] Sheahan T. P., Sims A. C., Graham R. L., Menachery V. D., Gralinski L. E., Case J. B., Leist S. R., Pyrc K., Feng J. Y., Trantcheva I., Bannister R., Park Y., Babusis D., Clarke M. O., Mackman R. L., et al (2017) Broad-spectrum antiviral GS-5734 inhibits both epidemic and zoonotic coronaviruses. Sci. Transl. Med. 9, eaal3653 10.1126/scitranslmed.aal3653 28659436 PMC5567817

[12] Pruijssers A. J., George A. S., Schäfer A., Leist S. R., Gralinksi L. E., Dinnon K. H. 3rd, Yount B. L., Agostini M. L., Stevens L. J., Chappell J. D., Lu X., Hughes T. M., Gully K., Martinez D. R., Brown A. J., et al (2020) Remdesivir inhibits SARS-CoV-2 in human lung cells and chimeric SARS-CoV expressing the SARS-CoV-2 RNA polymerase in mice. Cell Rep. 32, 107940 10.1016/j.celrep.2020.107940 32668216 PMC7340027

[13] Gordon C. J., Tchesnokov E. P., Feng J. Y., Porter D. P., and Götte M. (2020) The antiviral compound remdesivir potently inhibits RNA-dependent RNA polymerase from Middle East respiratory syndrome coronavirus. J. Biol. Chem. 295, 4773–4779 10.1074/jbc.AC120.013056 32094225 PMC7152756

[14] Gordon C. J., Tchesnokov E. P., Woolner E., Perry J. K., Feng J. Y., Porter D. P., and Götte M. (2020) Remdesivir is a direct-acting antiviral that inhibits RNA-dependent RNA polymerase from severe acute respiratory syndrome coronavirus 2 with high potency. J. Biol. Chem. 295, 6785–6797 10.1074/jbc.RA120.013679 32284326 PMC7242698

[15] Traut T. W. (1994) Physiological concentrations of purines and pyrimidines. Mol. Cell. Biochem. 140, 1–22 10.1007/BF00928361 7877593

[16] Kennedy E. M., Gavegnano C., Nguyen L., Slater R., Lucas A., Fromentin E., Schinazi R. F., and Kim B. (2010) Ribonucleoside triphosphates as substrate of human immunodeficiency virus type 1 reverse transcriptase in human macrophages. J. Biol. Chem. 285, 39380–39391 10.1074/jbc.M110.178582 20924117 PMC2998149

[17] Minskaia E., Hertzig T., Gorbalenya A. E., Campanacci V., Cambillau C., Canard B., and Ziebuhr J. (2006) Discovery of an RNA virus 3'→5' exoribonuclease that is critically involved in coronavirus RNA synthesis. Proc. Natl. Acad. Sci. U. S. A. 103, 5108–5113 10.1073/pnas.0508200103 16549795 PMC1458802

[18] Ferron F., Subissi L., Silveira De Morais A. T., Le N. T. T., Sevajol M., Gluais L., Decroly E., Vonrhein C., Bricogne G., Canard B., and Imbert I. (2018) Structural and molecular basis of mismatch correction and ribavirin excision from coronavirus RNA. Proc. Natl. Acad. Sci. U. S. A. 115, E162–E171 10.1073/pnas.1718806115 29279395 PMC5777078

[19] Bouvet M., Imbert I., Subissi L., Gluais L., Canard B., and Decroly E. (2012) RNA 3′-end mismatch excision by the severe acute respiratory syndrome coronavirus nonstructural protein nsp10/nsp14 exoribonuclease complex. Proc. Natl. Acad. Sci. U. S. A. 109, 9372–9377 10.1073/pnas.1201130109 22635272 PMC3386072

[20] Subissi L., Posthuma C. C., Collet A., Zevenhoven-Dobbe J. C., Gorbalenya A. E., Decroly E., Snijder E. J., Canard B., and Imbert I. (2014) One severe acute respiratory syndrome coronavirus protein complex integrates processive RNA polymerase and exonuclease activities. Proc. Natl. Acad. Sci. U. S. A. 111, E3900–E3909 10.1073/pnas.1323705111 25197083 PMC4169972

[21] Kirchdoerfer R. N., and Ward A. B. (2019) Structure of the SARS-CoV nsp12 polymerase bound to nsp7 and nsp8 co-factors. Nat. Commun. 10, 2342 10.1038/s41467-019-10280-3 31138817 PMC6538669

[22] Hillen H. S., Kokic G., Farnung L., Dienemann C., Tegunov D., and Cramer P. (2020) Structure of replicating SARS-CoV-2 polymerase. Nature 584, 154–156 10.1038/s41586-020-2368-8 32438371

[23] Wang Q., Wu J., Wang H., Gao Y., Liu Q., Mu A., Ji W., Yan L., Zhu Y., Zhu C., Fang X., Yang X., Huang Y., Gao H., Liu F., et al (2020) Structural basis for RNA replication by the SARS-CoV-2 polymerase. Cell 182, 417–428e413 10.1016/j.cell.2020.05.034 32526208 PMC7242921

[24] Peng Q., Peng R., Yuan B., Zhao J., Wang M., Wang X., Wang Q., Sun Y., Fan Z., Qi J., Gao G. F., and Shi Y. (2020) Structural and biochemical characterization of the nsp12-nsp7-nsp8 core polymerase complex from SARS-CoV-2. Cell Rep. 31, 107774 10.1016/j.celrep.2020.107774 32531208 PMC7260489

[25] Wiersinga W. J., Rhodes A., Cheng A. C., Peacock S. J., and Prescott H. C. (2020) Pathophysiology, transmission, diagnosis, and treatment of coronavirus disease 2019 (COVID-19): A Review. JAMA 324, 782–793 10.1001/jama.2020.12839 32648899

[26] Tchesnokov E. P., Obikhod A., Schinazi R. F., and Götte M. (2008) Delayed chain termination protects the anti-hepatitis B virus drug entecavir from excision by HIV-1 reverse transcriptase. J. Biol. Chem. 283, 34218–34228 10.1074/jbc.M806797200 18940786 PMC2590697

[27] Gordon C. J., Tchesnokov E. P., Feng J. Y., Porter D. P., and Götte M. (2020) The antiviral compound remdesivir potently inhibits RNA-dependent RNA polymerase from Middle East respiratory syndrome coronavirus. J. Biol. Chem. 295, 4773–4779 10.1074/jbc.AC120.013056 32094225 PMC7152756

[28] Marchand B., and Götte M. (2003) Site-specific footprinting reveals differences in the translocation status of HIV-1 reverse transcriptase. Implications for polymerase translocation and drug resistance. J. Biol. Chem. 278, 35362–35372 10.1074/jbc.M304262200 12819205

[29] Seifert M., Bera S. C., van Nies P., Kirchdoerfer R. N., Shannon A., Le T.-T.-N., Grove T. L., Papini F. S., Arnold J. J., Almo S. C., Canard B., Depken M., Cameron C. E., and Dulin D. (2020) Signatures and mechanisms of efficacious therapeutic ribonucleotides against SARS-CoV-2 revealed by analysis of its replicase using magnetic tweezers. bioRxiv 2020

[30] Dulin D., Arnold J. J., van Laar T., Oh H. S., Lee C., Perkins A. L., Harki D. A., Depken M., Cameron C. E., and Dekker N. H. (2017) Signatures of nucleotide analog incorporation by an RNA-dependent RNA polymerase revealed using high-throughput magnetic tweezers. Cell Rep. 21, 1063–1076 10.1016/j.celrep.2017.10.005 29069588 PMC5670035

[31] Magee W. C., Aldern K. A., Hostetler K. Y., and Evans D. H. (2008) Cidofovir and (S)-9-[3-hydroxy-(2-phosphonomethoxy)propyl]adenine are highly effective inhibitors of vaccinia virus DNA polymerase when incorporated into the template strand. Antimicrob. Agents Chemother. 52, 586–597 10.1128/AAC.01172-07 18056278 PMC2224733

[32] Maag D., Castro C., Hong Z., and Cameron C. E. (2001) Hepatitis C virus RNA-dependent RNA polymerase (NS5B) as a mediator of the antiviral activity of ribavirin. J. Biol. Chem. 276, 46094–46098 10.1074/jbc.C100349200 11602568

[33] Tchesnokov E. P., Feng J. Y., Porter D. P., and Götte M. (2019) Mechanism of inhibition of Ebola virus RNA-dependent RNA polymerase by remdesivir. Viruses 11, 326 10.3390/v11040326 30987343 PMC6520719

[34] Tchesnokov E. P., Raeisimakiani P., Ngure M., Marchant D., and Götte M. (2018) Recombinant RNA-dependent RNA polymerase complex of Ebola virus. Sci. Rep. 8, 3970 10.1038/s41598-018-22328-3 29507309 PMC5838098

