# Correction: Template-dependent inhibition of coronavirus RNA-dependent RNA polymerase by remdesivir reveals a second mechanism of action

**DOI:** 10.1016/j.jbc.2021.101048

**Published:** 2021-08-06

**Authors:** Egor P. Tchesnokov, Calvin J. Gordon, Emma Woolner, Dana Kocincova, Jason K. Perry, Joy Y. Feng, Danielle P. Porter, Matthias Götte

VOLUME 295 (2020), PAGES 16156–16165

The name of the fourth coauthor of this article was misspelled. It should read “Dana Kocincova” and not “Dana Kocinkova.”

