# Supplementary material for: Template-dependent inhibition of coronavirus RNA-dependent RNA polymerase by remdesivir reveals a second mechanism of action
Source: J Biol Chem. 2020 Sep 23;295(47):16156–65. doi: 10.1074/jbc.AC120.015720 (PMC7681019; doi:10.1074/jbc.AC120.015720)
Supplement: Supporting Information [file supp_295_47_16156__index.html]

Template-dependent inhibition of coronavirus RNA-dependent RNA polymerase by remdesivir reveals a second mechanism of action — Second Mechanism of Remdesivir — Template-dependent inhibition of coronavirus RNA-dependent RNA polymerase by remdesivir reveals a second mechanism of action — Second mechanism of remdesivir — Supporting Information 

# Template-dependent inhibition of coronavirus RNA-dependent RNA polymerase by remdesivir reveals a second mechanism of action

## Supporting Information

- Supporting Information (to be published online) - SI Tables and Figures
