# Supplementary material for: Template-dependent inhibition of coronavirus RNA-dependent RNA polymerase by remdesivir reveals a second mechanism of action
Source: J Biol Chem. 2020 Sep 23;295(47):16156–65. doi: 10.1074/jbc.AC120.015720 (PMC7681019; doi:10.1074/jbc.AC120.015720)
Supplement: Supporting Information [file supp_AC120.015720_163031_1_supp_594398_qgd1ls.pdf]

Supporting information for

# Template-dependent inhibition of coronavirus RNA-dependent RNA polymerase by remdesivir reveals a second mechanism of action

**Egor P. Tchesnokov[1#], Calvin J. Gordon[1#], Emma Woolner[1], Dana Kocinkova[1], Jason K. Perry[3], Joy Y. Feng[3], Danielle P. Porter[3] and Matthias Götte[1,2*]**

From the [1] Department of Medical Microbiology and Immunology, University of Alberta, Edmonton, Alberta, Canada; [2] Li Ka Shing Institute of Virology at University of Alberta, Edmonton, Alberta, Canada; [3] Gilead Sciences, Inc., Foster City, California, USA

* To whom correspondence should be addressed: Matthias Götte: Department of Medical Microbiology and Immunology, University of Alberta, Edmonton, Alberta, Canada, T6G 2E1; gotte@ualberta.ca; Tel.1(780) 492-2308.
[#] EPT and CJG contributed equally to this work.

## List of included material

- **Figure S1.** Expression construct, SDS PAGE and mass spectroscopic analyses of the SARS-CoV-2 RdRp complexes.
- additional information on cloning, expression and purification of SARS-CoV-2 RdR complexes
- **Figure S2.** Quality of the RNA templates produced by T7 RNA polymerase.
- **Table S1.** UTP and ATP relative efficiency of incorporation opposite or after remdesivir in the template, respectively, catalyzed by SARS-CoV-2 wild-type and V557L mutant RdRp complexes.
- **Figure S3.** RNA synthesis catalyzed by SARS-CoV-2 RdRp wild type and V557L complexes on an RNA template containing remdesivir embedded in the template.
- **Table S2.** Remdesivir-TP (RDV-TP) selectivity values of for incorporation opposite U in the template catalyzed by SARS-CoV-2 wild-type and V557L mutant RdRp complexes.
- **Figure S4.** RNA synthesis catalyzed by SARS-CoV-2 RdRp wild-type and V557L mutant complexes on an RNA template containing multiple or a single site for RDV-TP incorporation.
- **Figure S5.** RNA synthesis catalyzed by SARS-CoV-2 wild type and S861G mutant RdRp complexes.

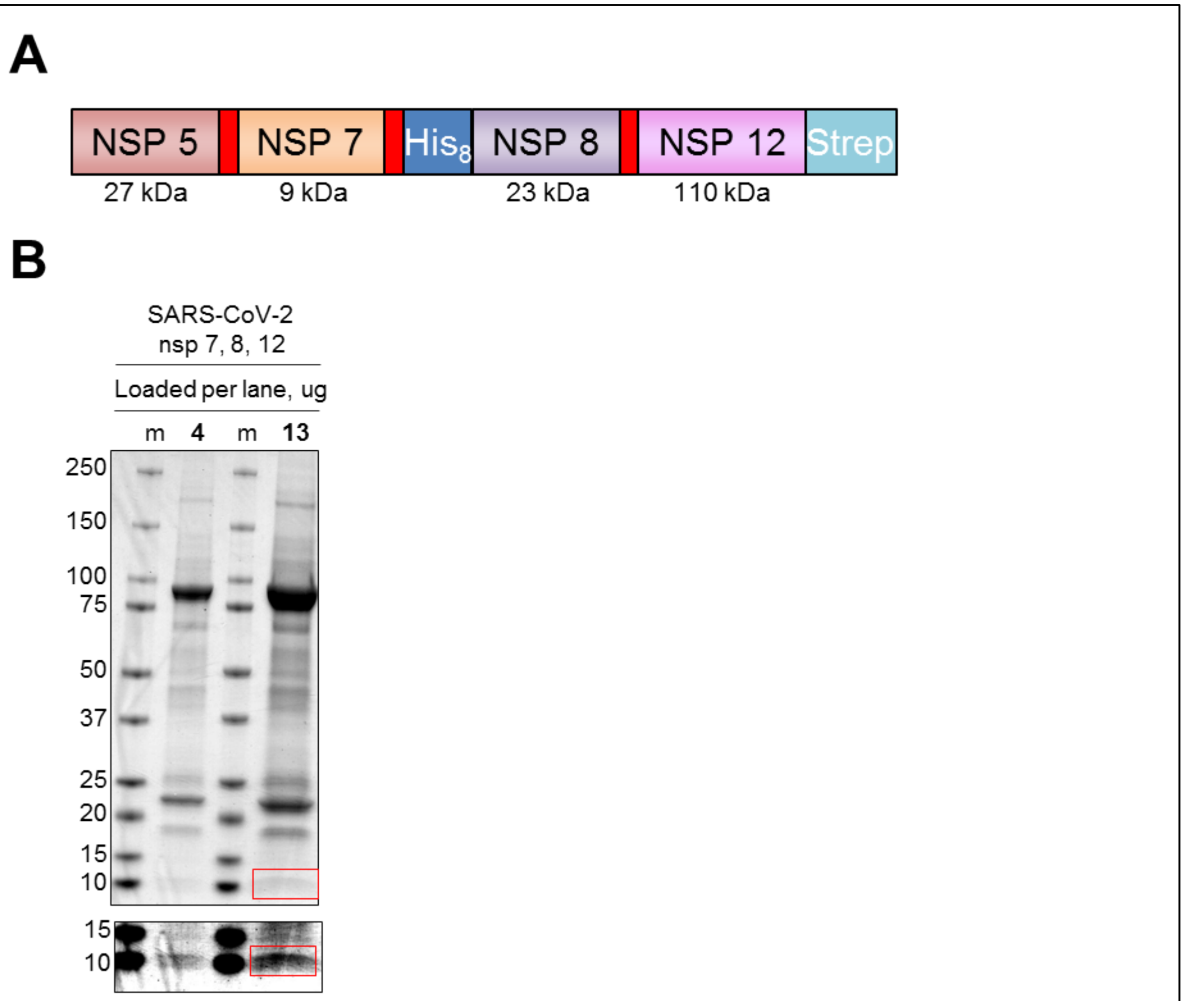


**C**

| Accession | Description | Score | Coverage | # Proteins | # Unique Peptides | # Peptides |
|---|---|---|---|---|---|---|
| sp\|7 | sp\|7 | 30.53 | 84.34 | 1 | 6 | 6 |
| | A3 | Sequence | # PSMs | # Proteins | # Protein Groups | Protein Group Accessions |
| | High | LWAQcVQLHNDILLAK | 3 | 1 | 1 | sp\|7 |
| | High | cTSVVLLSVLQQLR | 4 | 1 | 1 | sp\|7 |
| | High | LcEEmLDNR | 12 | 1 | 1 | sp\|7 |
| | High | LcEEmLDnRATLQ | 2 | 1 | 1 | sp\|7 |
| | High | LcEEMLDNR | 2 | 1 | 1 | sp\|7 |
| | High | DTTEAFEK | 4 | 1 | 1 | sp\|7 |
| | High | mVSLLSVLLSMQGAVDINK | 1 | 1 | 1 | sp\|7 |
| | High | mVSLLSVLLSmQGAVDINK | 1 | 1 | 1 | sp\|7 |
| | High | LWAQcVQLHnDILLAK | 6 | 1 | 1 | sp\|7 |

**Figure S1.** Expression construct, SDS PAGE and mass spectroscopic analyses of the SARS-CoV-2 RdRp complexes. (**A**) Schematic representaion of the expression construct (pFastBac1/nsp5-7-8-12) used to produce SARS-CoV-2 nsp7/nsp8/nsp12 RdRp complex. Genes coding for non-structural proteins, molecular weight of the expressed proteins in kDa and affinity

tags ($His_8$ and Strep, histidine and strep tags, respectively) are indicated. Red rectangles indicate the nsp5 protease cognate cleavage sites. (**B**) SDS PAGE migration pattern of the purified enzyme preparations stained with Coomassie Brilliant Blue G-250 dye. Bands migrating at ~100 kDa and ~25 kDa contain nsp12 and nsp8, respectively. Red rectangle defines the location on the gel which was submitted to the mass spectroscopic analysis. Bottom subpanel illustrates the portion of the gel where nsp7 is expected to migrate; in order to facilitate the visualization the contrast of the image was uniformly increased. (**C**) A snapshot of the mass spectroscopy data file of the portion of the gel illustrated by the red rectangle in panel B.

**Additional information on cloning, expression and purification of SARS-CoV-2 RdR complexes**

The pFastBac-1 (Invitrogen, Burlington, ON, Canada) plasmid with the codon-optimized synthetic DNA sequence (GenScript, Piscataway, NJ, USA) coding for a portion of 1ab polyprotein of SARS-CoV-2 (NCBI: QHD43415.1) containing only nsp5, nsp7, nsp8 and nsp12 (wild type or nsp12 V557L, S861G, S861A, S861P individual substitutions) was used as a starting material for protein expression in insect cells (Sf9, Invitrogen, Burlington, ON, Canada). The nsp5, nsp7, nsp8 and nsp12 were expressed in insect cells as a polyprotein containing the cognate nsp5 protease cleavage sites. We employed the MultiBac (Geneva Biotech, Indianapolis, IN, USA) system for protein expression in insect cells (Sf9, Invitrogen, Burlington, ON, Canada) according to published protocols (1,2). SARS-CoV-2 protein complexes were purified using Ni-NTA affinity chromatography of the nsp8 N-terminal eight-histidine tag according to the manufacturer's specifications (Thermo Scientific, Rockford, IL, USA).

The lysis buffer contained 100 Tris-HCl (pH 8), 100 mM KCl, 5 mM TCEP, 0.1% Tween-20, 250 mM sucrose, 25 mM imidazole and one tablet of protease inhibitors (Complete ULTRA, Mini, EDTA-free, Roche, Mannheim, Germany). Lysed cell pellet from 1L cell culture (~2 $*10^6$ cells/mL) were centrifuged at 1000*g for 25 minutes at 4˚C, supernatant was removed, solid NaCl was added to 1000 mM and centrifuged again at 30000*g for 15 minutes at 4˚C. The resulting supernatant was aliquoted on four 0.5 mL Ni-NTA columns and allowed to rotate for 1 hour at 4˚C. The columns were subsequently washed with 40 column volumes (CV) of a buffer containing 100 Tris-HCl (pH 8), 1000 mM NaCl, 5 mM TCEP, 0.01 % Tween-20, 10% glycerol, and 25 mM imidazole and with 6 CV of the same buffer but containing 50 mM imidazole. Proteins were eluted with the same buffer but containing 200 mM imidazole. Purified protein preparations were adjusted with glycerol to 40 % and stored at -20˚C. More than three independent protein preparations were used during the course of the study. SARS-CoV-2 wild type proteins as well as nsp12 mutants were confirmed by mass spectrometry (MS) analysis (Dr. Jack Moore, Alberta Proteomics and Mass Spectrometry, Edmonton, AB, Canada). The expression plasmid pFastBac1/nsp5-7-8-12 is available to the research community upon request.

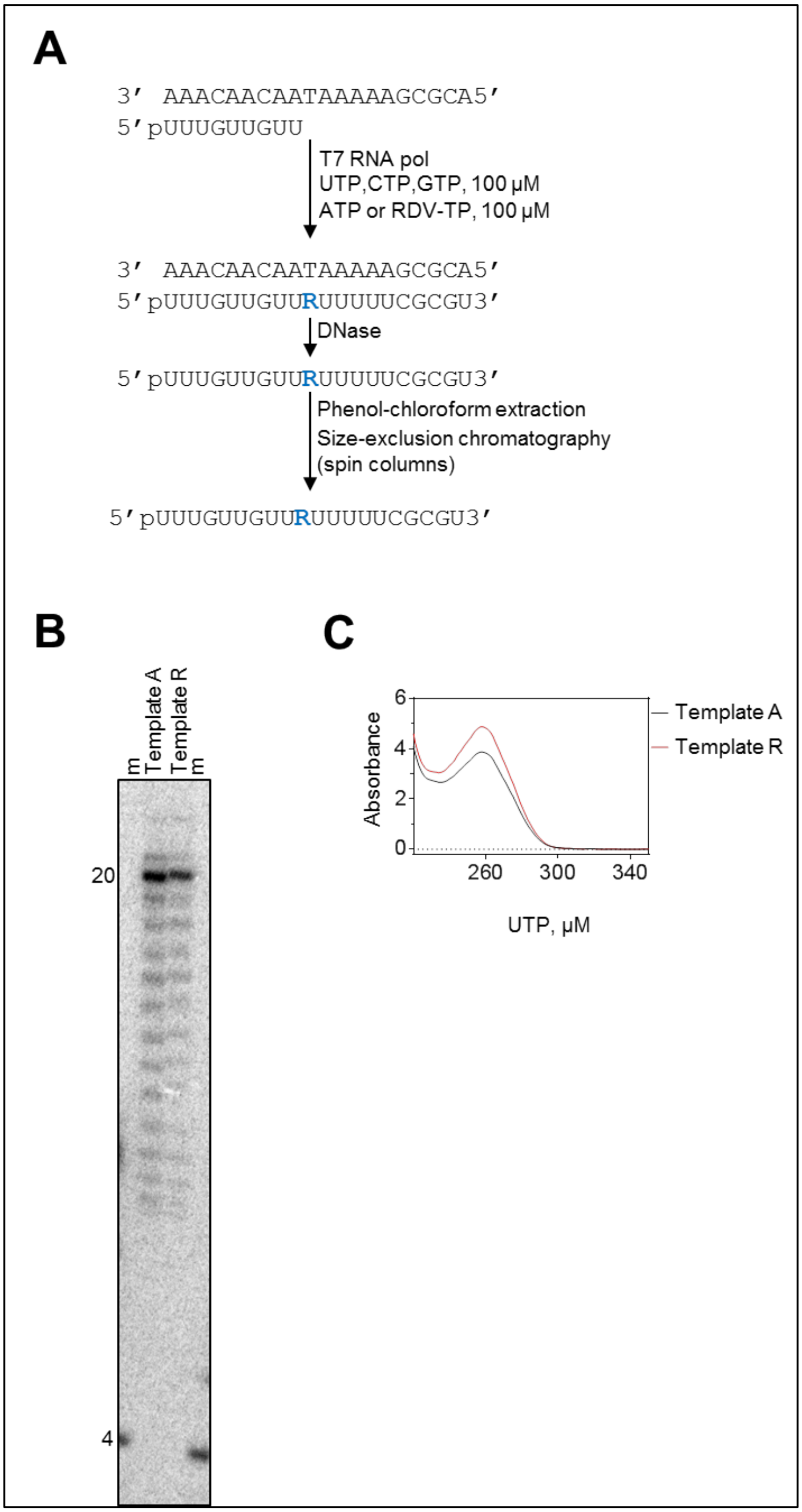


**Figure S2.** Quality of the RNA templates produced by T7 RNA polymerase. (**A**) Schematic representation of the T7 RNA polymerase reaction used to produce an RNA template with template-embedded RDV (R). (**B**) Migration pattern of RNA template preparations (after

phenol/chloroform extraction and spin-column size-exclusion chromatography) subjected to denaturing 8M urea PAGE. Trace amounts of [α-$^{32}$P]-CTP were added to the T7 RNA polymerase reactions to allow the visualization of the final reaction products. Template A and R refers to T7 RNA polymerase reaction conditions where UTP/CTP/GTP nucleotide cocktail was supplemented with ATP or RDV-TP, respectively. Full template-length product (20) fraction in Template A preparation is 1.2-fold higher than in Template R preparation. Template A and R exhibit identical and uniform RNA degradation products with the full template-length (20) product containing the strongest signal in the lane. These RNA template preparations were used only for the illustration of their migration pattern; they were not used in reactions with SARS-CoV-2 RdRp complex. 4 indicates the migration pattern of 5'-$^{32}$P-labeled 4-nt primer used here as a marker (m). (**C**) Example of an absorbance spectra of the RNA template preparations used in the reactions with SARS-CoV-2 RdRp complex after phenol/chloroform extraction and spin-column size-exclusion chromatography. These RNA templates were synthesized by T7 RNA polymerase using ATP/UTP/CTP/GTP or RDV-TP/ UTP/CTP/GTP cocktails in the absence of [α-$^{32}$P]-CTP. The a260 absorbance of Template R is 1.3-fold higher than of Template A.

**Table S1.** UTP and ATP relative efficiency of incorporation opposite or after remdesivir in the template, respectively, catalyzed by SARS-CoV-2 wild-type and V557L mutant RdRp complexes.

| | | |
|---|---|---|
| **Incorporation opposite RDV in the template** | | |
| | | UTP |
| **wild type** | $V_{max}$[a] (product fraction) | 0.76[d] ±0.010[e] |
| | $K_m$[b], μM | 3.6 ±0.24 |
| | $V_{max}/K_m$[c] | 0.21 |
| | | |
| **V557L** | $V_{max}$ (product fraction) | 0.67 ±0.019 |
| | $K_m$ , μM | 0.64 ±0.13 |
| | $V_{max}/K_m$ | 1.0 |
| | $\frac{\mathbf{V557L}\ (\frac{Vmax}{Km})}{\mathbf{wild\ type}\ (\frac{Vmax}{Km})}$ | **5** |
| | | |
| **Incorporation after RDV in the template** | | |
| | | ATP |
| **wild type** | $V_{max}$ (product fraction) | 0.31 ±0.0021 |
| | $K_m$ , μM | 2.1 ±0.068 |
| | $V_{max}/K_m$ | 0.15 |
| | | |
| **V557L** | $V_{max}$ (product fraction) | 0.33 ±0.013 |
| | $K_m$ , μM | 1.9 ±0.037 |
| | $V_{max}/K_m$ | 0.17 |
| | $\frac{\mathbf{V557L}\ (\frac{Vmax}{Km})}{\mathbf{wild\ type}\ (\frac{Vmax}{Km})}$ | **1.2** |
| | | |

[a] $V_{max}$ is a Michaelis–Menten parameter reflecting the maximal velocity of nucleotide incorporation.
[b] $K_m$ is a Michaelis–Menten parameter reflecting the concentration of the nucleotide substrate at which the velocity of nucleotide incorporation is half of $V_{max}$.
[c] Efficiency of nucleotide incorporation.
[d] All reported values have been calculated on the basis of a 8- or 10-data point experiments.
[e] Standard error associated with the fit.

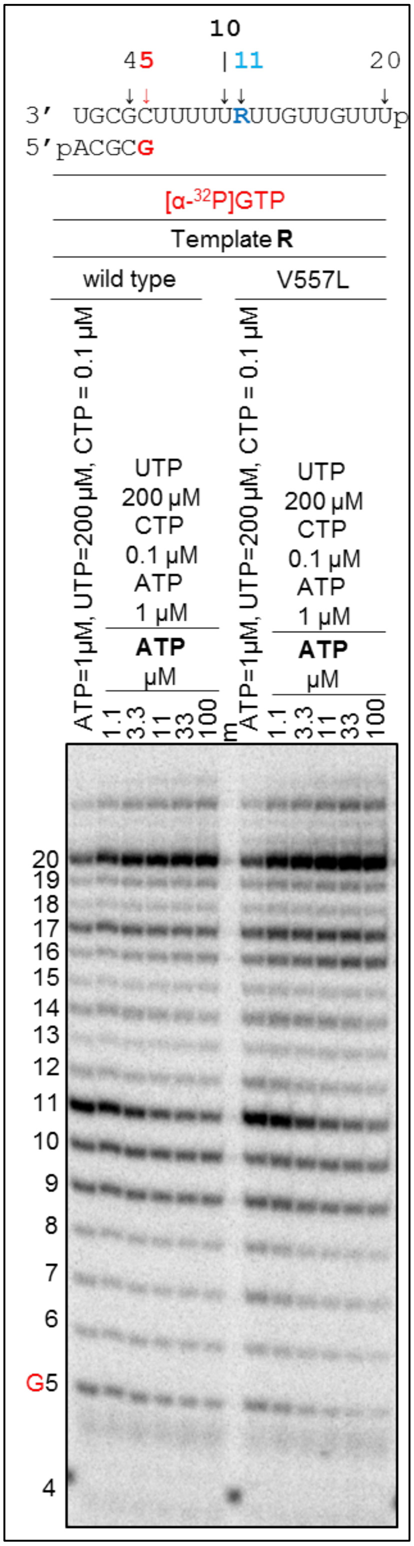


**Figure S3.** RNA synthesis catalyzed by SARS-CoV-2 RdRp wild type and V557L complexes on an RNA template containing remdesivir embedded in the template. RNA primer/template with template-embedded remdesivir (R) at position 11 is shown on top. G5 indicates the incorporation of [α-$^{32}$P]-GTP opposite template position 5. Below the primer/template sequence is the migration pattern of the products of RNA synthesis catalyzed by CoV-SARS-2 RdRp complexes

in the presence of RNA primer/template, $MgCl_2$, indicated concentrations of NTP cocktail supplemented with indicated concentrations of UTP after 30 minutes. 4 indicates the migration pattern of 5'-$^{32}$P-labeled 4-nt primer used here as a marker.

**Table S2.** Remdesivir-TP (RDV-TP) selectivity values of for incorporation opposite U in the template catalyzed by SARS-CoV-2 wild-type and V557L mutant RdRp complexes.

| Incorporation opposite U | | | |
|---|---|---|---|
| | | ATP | RDV-TP |
| **wild type** | $V_{max}$ [a] (product fraction) | 0.90[d] ±0.0081[f] | 0.87 ±0.018 |
| | $K_m$ [b], μM | 0.033 ±0.0012 | 0.013 ±0.00098 |
| | $V_{max}/K_m$ [c] | 27 | 67 |
| | **Selectivity, fold** [d] | | **0.41** |
| | | | |
| **V557L** | $V_{max}$ (product fraction) | 0.76 ±0.020 | 0.66 ±0.029 |
| | $K_m$ , μM | 0.091 ±0.012 | 0.033 ±0.0061 |
| | $V_{max}/K_m$ | 8.4 | 20 |
| | **Selectivity, fold** | | **0.42** |
| | | | |

[a] $V_{max}$ is a Michaelis–Menten parameter reflecting the maximal velocity of nucleotide incorporation.
[b] $K_m$ is a Michaelis–Menten parameter reflecting the concentration of the nucleotide substrate at which the velocity of nucleotide incorporation is half of $V_{max}$.
[c] Efficiency of nucleotide incorporation.
[d] Selectivity of a viral RNA polymerase for a nucleotide substrate analogue is calculated as the ratio of the $V_{max}/K_m$ values for NTP and NTP analogue, respectively.
[e] All reported values have been calculated on the basis of a 8- or 10-data point experiment.
[f] Standard error associated with the fit.

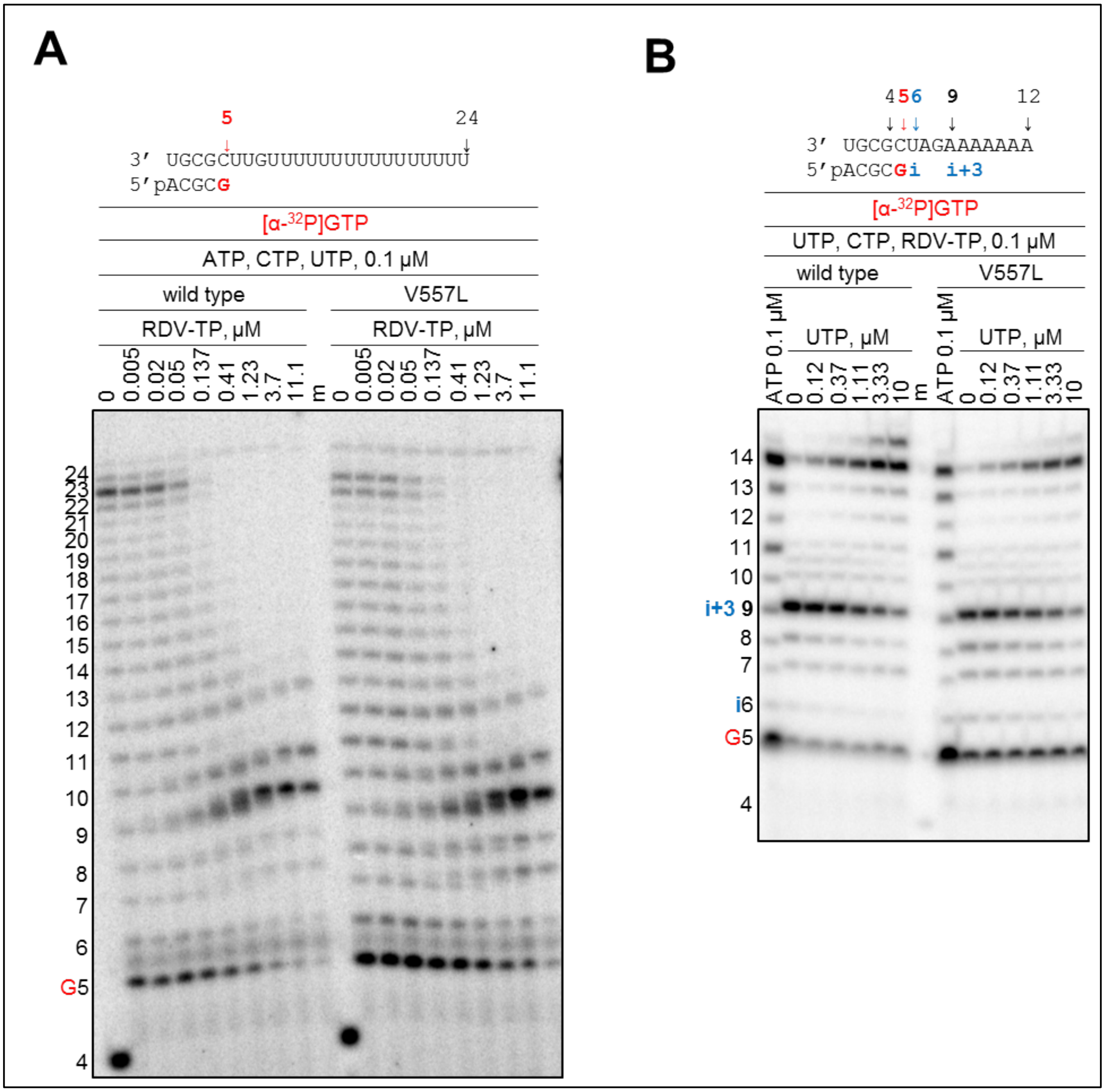


**Figure S4.** RNA synthesis catalyzed by SARS-CoV-2 RdRp wild-type and V557L mutant complexes on an RNA template containing multiple or a single site for RDV-TP incorporation. (**A**) Inhibition of RNA synthesis with increasing concentrations of RDV-TP. RNA primer/template supporting multiple incorporations of RDV-TP shown on top of the panel. G5 indicates the incorporation of [α-$^{32}$P]-GTP opposite template position 5. RNA synthesis was catalyzed by CoV-SARS-2 RdRp wild-type and mutant complexes in the presence of RNA primer/template, $MgCl_2$, indicated concentrations of NTP cocktail and increasing concentrations of RDV-TP for 30 minutes. “4” indicates the migration pattern of 5’-$^{32}$P-labeled 4-nt primer used here as a size marker. (**B**) Overcoming the delayed chain-termination at position i+3. RNA primer/template supporting incorporation of RDV-TP (i) at position 6 (i = 6) is shown on top. i+3 illustrates a delayed chain termination at position 9 when RDV-TP is incorporated at position 6. G5 indicates the incorporation of [α-$^{32}$P]-GTP opposite template position 5. RNA synthesis

was catalyzed by CoV-SARS-2 RdRp wild type and mutant complexes in the presence of RNA primer/template, $MgCl_2$ and indicated concentrations of NTP supplemented with increasing concentrations of UTP for 30 minutes. “4” indicates the migration pattern of 5’-$^{32}$P-labeled 4-nt primer used here as a size marker.

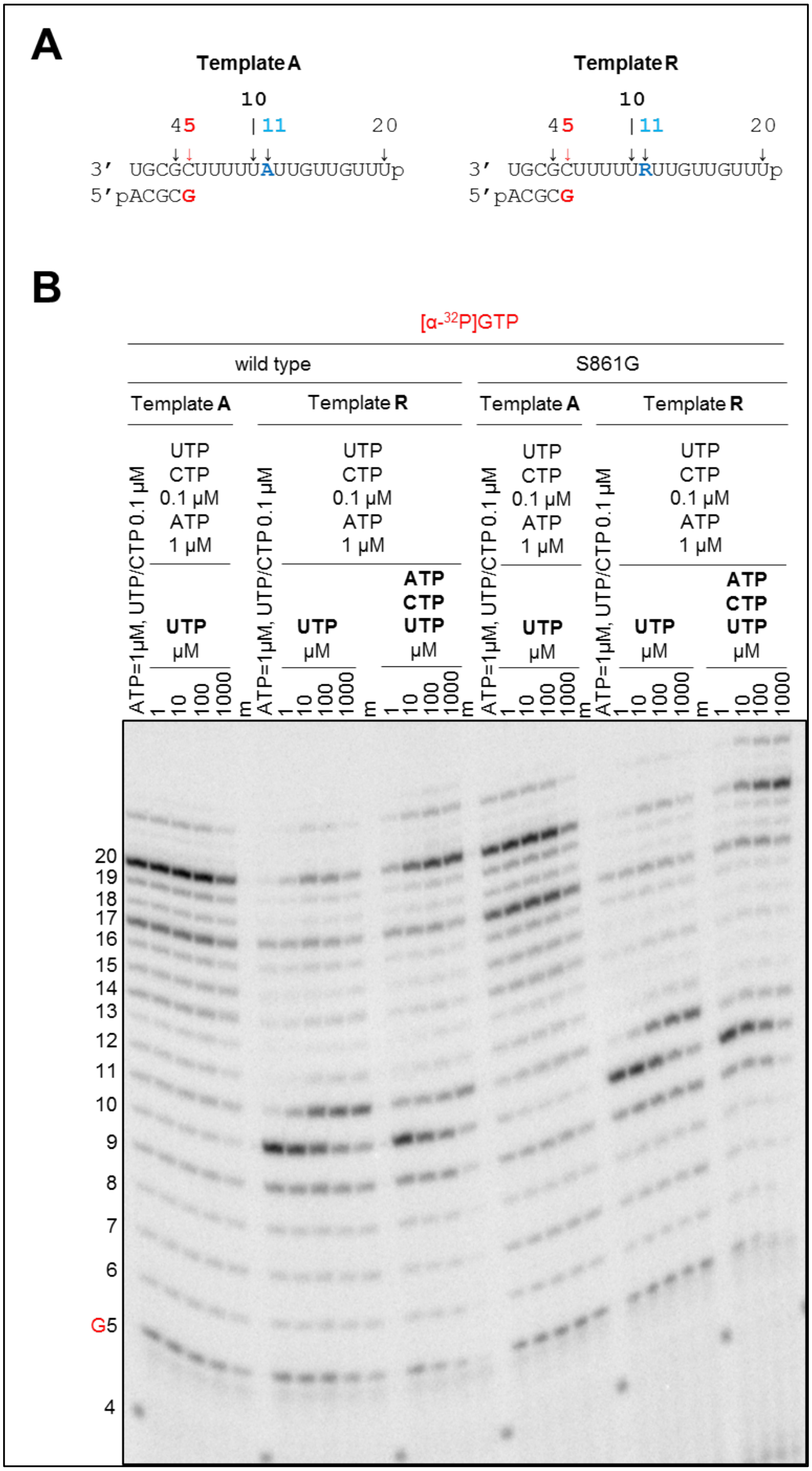


**Figure S5.** RNA synthesis catalyzed by SARS-CoV-2 wild type and S861G mutant RdRp complexes. (**A**) RNA primer/template with template-embedded RDV (Template R) at

position 11; the corresponding primer/template with adenosine at this position (Template A) is shown on the left. G5 indicates the incorporation of [α-$^{32}$P]-GTP opposite template position 5. (**B**) Migration pattern of the products of RNA synthesis catalyzed by CoV-SARS-2 RdRp wildtype and S861G mutant RdRp complexes in the presence of RNA primer/templates shown in panel A, $MgCl_2$ and indicated concentrations of NTP cocktails after 30 minutes. “4” indicates the migration pattern of 5’-$^{32}$P-labeled 4-nt primer used here as a size marker.


1. Berger, I., Fitzgerald, D. J., and Richmond, T. J. (2004) Baculovirus expression system for heterologous multiprotein complexes. *Nature biotechnology* **22**, 1583-1587
2. Bieniossek, C., Richmond, T. J., and Berger, I. (2008) MultiBac: multigene baculovirus-based eukaryotic protein complex production. *Current protocols in protein science / editorial board, John E. Coligan ... [et al.]* **Chapter 5**, Unit 5 20
